# Short-term plasticity and modulation of synaptic transmission at mammalian inhibitory cholinergic olivocochlear synapses

**DOI:** 10.3389/fnsys.2014.00224

**Published:** 2014-12-02

**Authors:** Eleonora Katz, Ana Belén Elgoyhen

**Affiliations:** ^1^Instituto de Investigaciones en Ingeniería Genética y Biología Molecular “Dr. Héctor N. Torres” (INGEBI), Consejo Nacional de Investigaciones Científicas y Técnicas (CONICET)Buenos Aires, Argentina; ^2^Departamento de Fisiología, Biología Molecular y Celular “Prof. Héctor Maldonado”, Facultad de Ciencias Exactas y Naturales, Universidad de Buenos AiresBuenos Aires, Argentina; ^3^Tercera Cátedra de Farmacología, Facultad de Medicina, Universidad de Buenos AiresBuenos Aires, Argentina

**Keywords:** medial olivocochlear system, efferent innervation, cochlear hair cells, synaptic transmission, calcium channels, calcium-activated potassium channels, GABA_B_ receptors, short-term synaptic plasticity

## Abstract

The organ of Corti, the mammalian sensory epithelium of the inner ear, has two types of mechanoreceptor cells, inner hair cells (IHCs) and outer hair cells (OHCs). In this sensory epithelium, vibrations produced by sound waves are transformed into electrical signals. When depolarized by incoming sounds, IHCs release glutamate and activate auditory nerve fibers innervating them and OHCs, by virtue of their electromotile property, increase the amplification and fine tuning of sound signals. The medial olivocochlear (MOC) system, an efferent feedback system, inhibits OHC activity and thereby reduces the sensitivity and sharp tuning of cochlear afferent fibers. During neonatal development, IHCs fire Ca^2+^ action potentials which evoke glutamate release promoting activity in the immature auditory system in the absence of sensory stimuli. During this period, MOC fibers also innervate IHCs and are thought to modulate their firing rate. Both the MOC-OHC and the MOC-IHC synapses are cholinergic, fast and inhibitory and mediated by the α9α10 nicotinic cholinergic receptor (nAChR) coupled to the activation of calcium-activated potassium channels that hyperpolarize the hair cells. In this review we discuss the biophysical, functional and molecular data which demonstrate that at the synapses between MOC efferent fibers and cochlear hair cells, modulation of transmitter release as well as short term synaptic plasticity mechanisms, operating both at the presynaptic terminal and at the postsynaptic hair-cell, determine the efficacy of these synapses and shape the hair cell response pattern.

## Introduction

The organ of Corti, the mammalian sensory epithelium of the inner ear, has two types of mechanoreceptor cells, inner hair cells (IHCs) and outer hair cells (OHCs). In this sensory epithelium, vibrations produced by sound waves are transformed into electrical signals that depolarize the hair cell membranes (Hudspeth, 1997). Inner hair cells the phonoreceptors proper, release glutamate upon depolarization by incoming sounds and activate the auditory nerve fibers innervating them (Fuchs et al., 2003). Outer hair cells respond to variations in membrane voltage with changes in their length due to their electromotile property (Brownell et al., 1985). This enhances sound-evoked motion within the cochlear partition thereby amplifying the input to the IHCs. A descending efferent feedback pathway from the central nervous systems (CNS) reduces the sensitivity and sharp tuning of cochlear afferent fibers (Ashmore, 2008).

This efferent innervation is supplied by descending olivocochlear (OC) neurons (Rasmussen, 1955) and can be divided into two separate systems, namely the lateral and medial OC (MOC) systems (Warr, 1975, 1992; Guinan et al., 1983). In adult mammals, lateral OC (LOC) neurons whose somata are in the lateral superior olive (LSO) in the brainstem, project mainly to the ipsilateral cochlea and innervate the dendrites of spiral ganglion neurons (SGN) below the IHCs. Medial olivocochlear neurons, whose somata are in the medial periolivary region, project mostly (50–80% depending on species and cochlear region) to the contra lateral cochlea and make synaptic contacts with the OHC (Smith and Rasmussen, 1963; Liberman, 1980; Ginzberg and Morest, 1984; Liberman and Brown, 1986; Maison et al., 2003). The LOC and MOC systems are cholinergic but other neurotransmitters and neuromodulators like γ-aminobutiric acid (GABA), calcitonine gene-related peptide (CGRP) have also been found to be present in both types of OC fibers. In addition dopamine, and opiod peptides might also be expressed in LOC synaptic terminals (for an in depth description of the pharmacology and neurochemistry of the OC systems see Sewell, 2011). In rodents, the first efferent fibers can be traced toward sensory epithelia around embryonic day (E) 13 (Fritzsch and Nichols, 1993; Fritzsch, 1996). Medial olivocochlear efferent neurons mature early and project transiently to the IHC region of the cochlea before reaching their final targets, the OHCs (Simmons et al., 1996; Simmons, 2002). By postnatal day (P) 0, efferent axons make transient axo-somatic synapses with the IHCs and start to appear below the OHC area by P2, the first synapses are seen by P4 (Simmons et al., 1996; Bruce et al., 2000; Simmons, 2002; Rontal and Echteler, 2003). At around the onset of hearing, P12 in altricial rodents, axo-somatic synapses on IHCs have almost completely disappeared and only the axo-dendritc synapses between LOC fibers onto Type I SGN can be observed below the IHC area (Simmons et al., 1996). At this stage, OC innervation acquires the adult profile described above, where only OHCs present MOC axo-somatic synapses (Simmons, 2002).

### The medial olivocochlear system inhibits OHC activity

Medial olivocochlear neurons are activated by several feedback loops both from the periphery and cortical processing centers and regulate various aspects of auditory processing. Namely, the dynamic range of hearing (Guinan, 2011), the detection of relevant auditory signals (Maison et al., 2001), selective attention (Delano et al., 2007), and protection from noise-induced trauma (Rajan, 2000; Maison et al., 2002, 2013b; Taranda et al., 2009). When MOC fibers are activated by electrical stimuli, both sound-evoked movements in the cochlea and auditory nerve responses are reduced. This indicates that the MOC system reduces the gain of the cochlea by directly inhibiting OHC electromotile activity (Guinan, 2011). It has been demonstrated that the strength of cochlear inhibition is proportional to the firing frequency of MOC fibers (Galambos, 1956; Wiederhold and Kiang, 1970). Moreover, *in vitro* experiments using electrical stimulation suggest that the higher the rate of MOC activity, the higher the strength of synaptic inhibition at the MOC-OHC synapse (Ballestero et al., 2011).

### The MOC system inhibits IHC spontaneous activity before the onset of hearing

Altricial rodents are deaf at birth and start to hear at around P12. Before hearing, IHCs fire action potentials which result from the activation of an inward Ca^2+^ current and the slowly activating delayed rectifier potassium channel IK_neo_ (Kros et al., 1998; Marcotti et al., 2003b; Johnson et al., 2011). There is no consensus however regarding the origin of these calcium action potentials in immature IHCs, as it has also been suggested that they are evoked by ATP released from supporting cells present in the organ of Kolliker (Tritsch et al., 2007). Notwithstanding, these Ca^2+^ action potentials, whether spontaneous or evoked by ATP, release the neurotransmitter glutamate at the first auditory synapse in the absence of sensory stimuli (Beutner and Moser, 2001; Glowatzki and Fuchs, 2002) and promote activity in the immature auditory system (Johnson et al., 2011; Sendin et al., 2014) which might be involved in directing the first stages of central synapse formation (Kandler, 2004). As mentioned above, mature IHCs are mainly innervated by the dendrites of SGN afferent fibers. However, during early postnatal development, IHCs receive transient axo-somatic contacts from MOC efferent fibers, even before they reach their final targets, the OHCs (Simmons et al., 1996; Simmons, 2002). This innervation, like that on mature OHCs, is cholinergic and inhibitory (Glowatzki and Fuchs, 2000; Elgoyhen et al., 2001; Katz et al., 2004). From P1 to P12, when exogenous ACh is applied or when the efferent fibers are electrically stimulated (Glowatzki and Fuchs, 2000; Goutman et al., 2005), IHCs are hyperpolarized and consequently, Ca^2+^ action potential frequency is reduced or even abolished. Interestingly, just after birth, at P0, the MOC-IHC synapse was found to be excitatory and to increase action potential frequency in the IHCs (Roux et al., 2011). Therefore, it is likely that this transient innervation interferes with the generation of Ca^2+^ action potentials (Marcotti et al., 2003a, 2004; Johnson et al., 2011; Sendin et al., 2014) thereby modulating the release of glutamate which occurs in the absence of sensory stimulation before the onset of hearing (Beutner and Moser, 2001; Glowatzki and Fuchs, 2002).

## The MOC-hair cell synapse

### Molecular and functional properties of the postsynaptic response

Hair cells are inhibited by the MOC system in a few milliseconds, however, this MOC-hair cell synapses differ from other inhibitory fast synapses which are mediated by a chloride conductance through GABA and/or glycine receptors (Alger, 1991; Betz et al., 1999). Medial olivocochlear-hair cell synapses are mediated by the activation of nicotinic cholinergic receptors (nAChR) that mediate fast excitatory synaptic responses (Dani and Bertrand, 2007; Martyn et al., 2009). The cellular mechanisms of this cholinergic inhibition and the molecular constituents involved are common among vertebrates (Art et al., 1984; Fuchs, 1992; Erostegui et al., 1994a,b; Evans, 1996; Fuchs, 1996; Nenov et al., 1996; Glowatzki and Fuchs, 2000; Oliver et al., 2000; Katz et al., 2011). At the MOC-hair cell synapse inhibition is brought about by ACh acting on a cholinergic nicotinic receptor highly permeable to Ca^2+^, the α9α10 nAChR functionally coupled to the opening of Ca^2+^-activated K^+^ channels that hyperpolarize the OHC (Erostegui et al., 1994a; Evans, 1996; Nenov et al., 1996; Oliver et al., 2000; Wersinger et al., 2010; Ballestero et al., 2011; Wersinger and Fuchs, 2011). Pharmacological and immunohistochemical studies (Murthy et al., 2009a,b; Elgoyhen and Katz, 2012) together with molecular studies involving genetically modified mouse models (Vetter et al., 1999, 2007; Kong et al., 2008; Murthy et al., 2009a,b; Taranda et al., 2009) support this hypothesis.

**Figure 1:**
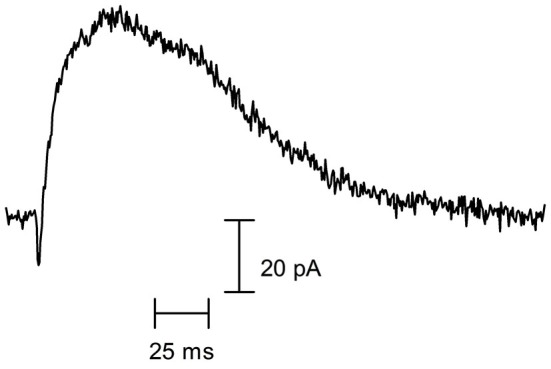
**Cholinergic synaptic currents of rodent cochlear hair cells**. Representative spontaneous synaptic current recorded in a P10 OHC from a mouse apical cochlear coil, voltage-clamped at −60 mV (recording performed by Jimena Ballestero). As can be observed, a rapid inward current (mediated by the α9α10 nAChR) is curtailed by a larger and longer-lasting outward current (mediated by the SK2 K^+^ channel).

**Figure 2:**
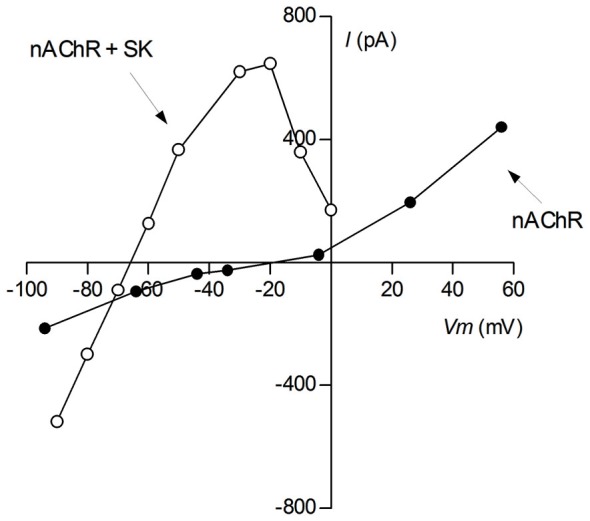
**Voltage sensitivity of cholinergic currents**. Ionic currents were evoked by bath application of 100 μM ACh to voltage-clamped P9-11 rat IHCs. Reprentative I-V curves of isolated cholinergic currents (nAChR; intracellular solution CsCl-BAPTA + 1 nM apamin) and of those coupled to the SK2 channel (nAChR + SK2; intracellular solution KCl-EGTA) (This I-V curve was reproduced with permission from Figure 1A in Gómez-Casati et al., 2005).

Inhibition in cochlear hair cells is brought about by a nicotinic receptor, the α9α10 nAChR, coupled to the activation of calcium-activated potassium channels, namely, SK2 type channels (Glowatzki and Fuchs, 2000; Oliver et al., 2000; Gómez-Casati et al., 2005; Elgoyhen and Katz, 2012; see Figures 1, 2) or BK channels (Wersinger et al., 2010; Wersinger and Fuchs, 2011). It has been described that when SK2 channels are blocked by apamin, a specific antagonist of these potassium channels, the cholinergic response at the cellular level changes from inhibitory to excitatory (Glowatzki and Fuchs, 2000; Katz et al., 2004; Marcotti et al., 2004; Gómez-Casati et al., 2005). Surprisingly, SK2 knockout mice lack these cholinergic excitatory efferent effects. The nAChRs are profoundly affected by deletion of the SK2 gene whereas this deletion does not affect voltage-gated conductances in the hair cells. SK2-knockout OHCs and neonatal IHCs are completely insensitive to exogenous ACh and lack efferent synaptic currents, implying absent or dysfunctional nAChRs (Kong et al., 2008). It has been therefore suggested that the SK2 channel might have a central role at these synapses and that the nAChR/SK2 channel complex is assembled before being inserted in the hair cell membrane (Kong et al., 2008). However, during normal development, the transient efferent innervation to the IHCs is excitatory at the first postnatal day, meaning that the α9α10 nAChR is functional prior to the functional expression of the SK2 channel and its coupling to the cholinergic response (Roux et al., 2011). From P2 until P12–14, a stage at which this synapse disappears, cholinergic responses are inhibitory and always coupled to the activation of the SK2 channel (Katz et al., 2004; Goutman et al., 2005; Roux et al., 2011). Therefore, the absence of cholinergic responses in SK null mice could be due to the fact that OC fiber degeneration is also observed in these mice (Kong et al., 2008; Murthy et al., 2009a). Thus, apart from the lack of the SK2 protein to stabilize the synaptic complex, the lack of innervation, as shown at the neuromuscular junction (Sanes and Lichtman, 1999) and the absence of cross talk between the pre- and the postsynapse could also lead to alteration/disruption of the molecular components necessary for a cholinergic response.

It was recently shown that large conductance, calcium and voltage-gated (BK) potassium channels expressed by the OHCs at the area of efferent contacts, are the basis for ACh-mediated hyperpolarization in higher frequency regions of the rat cochlea (Wersinger et al., 2010). This differs from rat cochlear low frequency regions or the chicken hearing organ, where hair cell hyperpolarization is served by SK potassium channels (Fuchs and Murrow, 1992; Glowatzki and Fuchs, 2000; Wersinger and Fuchs, 2011). The calcium affinity of BK channels is two orders of magnitude lower than that of SK channels, requiring higher calcium influx for activation (Fakler and Adelman, 2008). Therefore, the amount of calcium entering through the α9α10 nAChR must play a key role in cholinergic inhibition in different regions of the cochlear axis and also in different species. In agreement with this notion, it has been shown recently that the Ca^2+^ permeability of α9α10 nAChRs is not uniform across species (Lipovsek et al., 2012, 2014). The Ca^2+^ permeability in chicken α9α10 nAChRs is unexpectedly low and similar to that of heteromeric neuronal nAChRs whereas in rat α9α10 nAChRs is high and similar to that of homomeric α7- and α8-containing receptors (Sgard et al., 2002; Weisstaub et al., 2002; Gómez-Casati et al., 2005; Lipovsek et al., 2012, 2014). Therefore, the increased Ca^2+^ permeability in the mammalian lineage (Lipovsek et al., 2014) might have evolved to help activate low-Ca^2+^-affinity, high-conductance BK channels in mammalian basal OHCs, whereas Ca^2+^ influx provided by the non mammalian nAChR suffices to activate the high-Ca^2+^-affinity, low-conductance SK channels (Fakler and Adelman, 2008; Wersinger et al., 2010; Wersinger and Fuchs, 2011; Lipovsek et al., 2012, 2014). To test this hypothesis further, it would be interesting to evaluate whether there are variations in the nAChR Ca^2+^ permeability between apical (coupled to SK) and basal OHCs (coupled to BK). The role of BK channels has been recently evaluated *in vivo* by comparing MOC efferent-mediated inhibition in BK knockout mice with that of their wild-type littermates. It was found that, both BK and SK channel significantly contribute to the MOC-efferent inhibition along most of the cochlear axis, except in the apical 20% of the cochlea, where it is difficult to evaluate the effects *in vivo* (Maison et al., 2013a).

Cholinergic inhibition of hair cells, therefore, relies on a rise in postsynaptic calcium to activate calcium-dependent potassium channels, irrespective of whether the α9α10 nAChR is coupled either to the SK2 or to the BK channel or both. In addition, it has been postulated that inhibition involves the near-membrane postsynaptic cistern (Smith and Sjostrand, 1961; Saito, 1983; Fuchs, 2014; Fuchs et al., 2014). Since the synaptic cistern is co-extensive with the efferent terminals, lying only 14 nm apart from the postsynaptic membrane (Fuchs et al., 2014), it defines a restricted diffusion space that might play an important role in calcium kinetics. This synaptic cistern has been proposed to serve as a calcium store, similar to the sarcoplasmic reticulum that supports contraction in muscle. The participation of a calcium store is supported by the effects of ryanodine and other store-active agents (Sridhar et al., 1997; Evans et al., 2000; Lioudyno et al., 2004). Experiments *in vitro* performed in OHCs, show that caffeine, a store depleting compound, potentiates whereas ryanodine (a modulator of calcium induced-calcium release) and ciclopiazonic acid (an antagonist of the sarcoplasmic/endoplasmic reticulum calcium ATPase, SERCA) reduce the amplitude of synaptic ACh currents and also the amplitude of currents evoked by exogenously applied ACh (Evans et al., 2000; Lioudyno et al., 2004). Evaluation of the MOC-efferent effects in experiments performed *in vivo* show that cochlear perfusion with ryanodine, ciclopiazonic acid and thapsigargin (another SERCA antagonist), enhances the magnitude of the efferent effects (Sridhar et al., 1997). Even though the *in vitro* and *in vivo* results show differences in the effects of both ryanodine and store-active compounds in the magnitude and sign of their effects, they nevertheless suggest that cholinergic inhibition might be due to both influx of calcium from the extracellular space and calcium release from the synaptic cistern (Fuchs, 2014). However, the short time course of efferent synaptic currents (Oliver et al., 2000) and the voltage-dependence of ACh-evoked currents (Martin and Fuchs, 1992) suggest that the cholinergic response is due only to calcium influx. Very recently, it was proposed (Fuchs, 2014) that the cisterns could act both as a store that releases calcium or as a sink, a fixed buffer that absorbs calcium allowing the rapid decay of cholinergic currents (Glowatzki and Fuchs, 2000; Oliver et al., 2000; Katz et al., 2004; Gómez-Casati et al., 2005; Ballestero et al., 2011). Thus, depending on the degree of activity, and therefore on the amount of calcium accumulated in the cisterns, cholinergic inhibition would take place by calcium influx from the extracellular space or by a combination of calcium influx and calcium release from internal stores (Fuchs, 2014).

### Molecular and functional properties of transmitter release at MOC-hair cell synapses

#### Calcium channels coupled to ACh release at MOC synaptic terminals

Neurotransmitter release at fast synapses takes place when the action potential invades and depolarizes the synaptic terminal which promotes the activation of Ca^2+^ channels and the consequent increase in cytosolic Ca^2+^ (Katz and Miledi, 1969). The release of neurotransmitter is triggered by Ca^2+^ influx through presynaptic voltage-gated Ca^2+^ channels (VGCC; Katz and Miledi, 1969). In mammals, fast synaptic transmission at both central and peripheral synapses is mediated by multiple types of VGCCs, including N-type, P/Q type and R-type (Katz et al., 1997; Plant et al., 1998; Reid et al., 2003; Catterall and Few, 2008; Catterall, 2011). Voltage-gated Ca^2+^ channels are formed by at least four different subunits (α1, α2-δ, β, sometimes also γ). The existence of multiple pore-forming α1 subunits accounts for the biophysical and pharmacological diversity of VGCCs. So far, 10 different α1 genes have been found and they have been divided into three families: Ca_v_1.1 (α1S), Ca_v_1.2 (α1C), Ca_v_1.3 (α1D) and Ca_v_1.4 (α1F) all giving rise to L-type Ca^2+^ currents; Ca_v_2.1 (α1A), Ca_v_2.2 (α1B) and Ca_v_2.3 (α1E) giving rise to P/Q, N and R-type Ca^2+^ currents, respectively; and Ca_v_3.1 (α1G), Ca_v_3.2 (α1H) and Ca_v_3.3 (α1I) all giving rise to T-type currents (Catterall, 1998, 2011; Catterall and Few, 2008). In the mouse cochlea, the occurrence of the α1 subunits Ca_v_1.2 (L-type), Ca_v_1.3 (L-type) and Ca_v_2.3 (R-type) has been shown by PCR analysis (Green et al., 1996). In addition, the α1A (P/Q-type) and α1G (T-type) were also found, by analyzing the ion channel transcriptome, to be expressed in the mammalian inner ear (Gabashvili et al., 2007).

Transmitter release from the IHC ribbon synapse has been shown to be mediated by L-type currents (Platzer et al., 2000; Brandt et al., 2003) and that the Ca_v_1.3 subunit is the predominant α1 subunit in neonatal IHCs and OHCs (Platzer et al., 2000; Michna et al., 2003; Layton et al., 2005). Using immunocytochemical techniques, Waka et al. (2003), reported that from P2 to P14 the predominant VGCC type expressed by medial efferent fibers is the Ca_v_2.3, or R-type VGCC, whereas as of P14 onwards the predominant subunit is Ca_v_1.2, suggesting that L-type channels might be involved in the release of ACh from MOC efferent fibers in adult mice.

By using an electrophysiological and pharmacological approach in the acutely isolated cochlear mouse preparation at P9–11, it has been shown that ACh release at the efferent-IHC synapse is supported by both N (Ca_v_2.2) and P/Q-type (Ca_v_2.1) VGCCs (Zorrilla de San Martín et al., 2010). At different synapses as well as at different developmental stages, differences in the relative contribution of P/Q- and N-type VGCC to synaptic transmission have been reported (Iwasaki et al., 2000; Ishikawa et al., 2005). Moreover, is has been shown that transmitter release is more strongly dependent on the Ca^2+^ concentration for P/Q- than for N-type VGCCs. In cerebellar synapses, Ca^2+^ cooperativity is around 4 and 2.5 for P/Q- and N-type VGCCs, respectively (Mintz et al., 1995). At the MOC-IHC synapse, cooperativity is around 2.5 (Zorrilla de San Martín et al., 2010), suggesting that at the transient MOC-IHC synapse, at least two Ca^2+^ ions are necessary to trigger the release of one ACh vesicle (Dodge and Rahamimoff, 1967). Notwithstanding, in the above mentioned work, Ca^2+^ cooperativity was assayed without discriminating between the two types of VGCC that support release. Therefore, it would be interesting to study whether the N- or the P/Q-type VGCC is more efficiently coupled to the release machinery at the MOC-IHC synapse.

#### Inhibition of ACh release by L-type VGCC functionally coupled to BK channels

BK channel activation requires membrane depolarization and high intracellular Ca^2+^ (Fakler and Adelman, 2008). These two conditions are usually achieved during the release of neurotransmitter. Therefore, BK channels have been shown to accelerate the repolarizing phase of the action potential and thereby terminate the release process (Vergara et al., 1998; Fakler and Adelman, 2008). It has been reported that activation of BK channels requires Ca^2+^ influx through closely coupled L-type VGCCs (Storm, 1987; Lingle et al., 1996; Prakriya et al., 1996). BK channels have low Ca^2+^ affinity (Fakler and Adelman, 2008), therefore, the formation of macromolecular complexes between BK channels and VGCC is necessary for reliably activating BK channels by Ca^2+^ influx without affecting other Ca^2+^-dependent intracellular processes (Fakler and Adelman, 2008).

**Figure 3:**
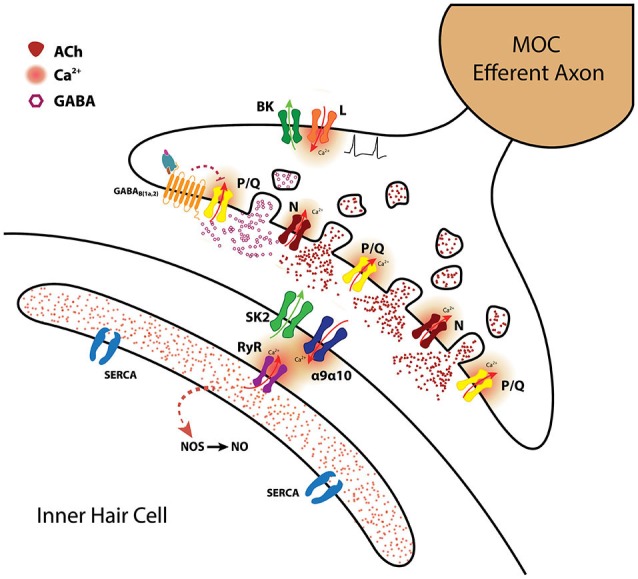
**The MOC-hair cell synapse**. Schematic representation of the molecules and mechanisms involved in synaptic transmission at MOC-hair cell synapses from the apical region of the organ of Corti. ACh release is supported by both P/Q and N-type VGCC. BK channels activated by Ca^2+^ entering through L-type VGCC accelerate action potential repolarization and thus diminish the amount of ACh being released (Zorrilla de San Martín et al., 2010). *γ*-aminobutiric acid probably co-released with ACh, activates presynaptic GABA_B(1a,2)_R which inhibit ACh release by altering the activity of P/Q-type VGCC (Wedemeyer et al., 2013). The inhibitory postsynaptic response is mediated by α9α10 nAChR coupled to the activation of SK2 K^+^ channels (Glowatzki and Fuchs, 2000; Elgoyhen et al., 2001; Katz et al., 2004; Gómez-Casati et al., 2005). Ca^2+^-induced Ca^2+^ release from the postsynaptic cisterns closely apposed to the IHC synaptic region probably also contributes to SK2 activation (Fuchs, 2014). Nitric oxide synthetized by the IHC in response to 1 Hz stimulation of the MOC fibers enhances transmitter release by the MOC synaptic terminals acting as a retrograde messenger (Kong et al., 2013). (SERCA: sarcoendoplasmic reticulum calcium ATPase; RyR: Ryanodine receptor; NOS: nitric oxide synthase; NO: nitric oxide) This drawing was kindly performed by Marcelo Moglie.

Before the onset of hearing, BK channels were shown by immunofluorescence to be present at the MOC synaptic terminals making axo-somatic contacts with the IHCs (Zorrilla de San Martín et al., 2010). Moreover, by electrophysiological recordings and the use of specific BK channel and L-type VGCC agonists and antagonists, it has been demonstrated that BK channels are functionally coupled to the activation of L-type VGCC (Zorrilla de San Martín et al., 2010). Those experiments show that when an action potential invades MOC synaptic terminals, P/Q-, N-, and L-type VGCCs are activated. Influx of Ca^2+^ via P/Q- and N-type VGCCs, closely associated to the release machinery, support release. Whereas, influx of Ca^2+^ via L-type VGCCs functionally associated to BK channels, and possibly farther away from the release machinery (Urbano et al., 2001; Flink and Atchison, 2003), together with membrane depolarization, would activate BK channels. BK channel activation would accelerate the repolarization of the MOC synaptic terminal membrane and ACh release would be reduced (Storm, 1987; Marcantoni et al., 2007) (this model is schematized in Figure 3).

It remains to be determined if, as reported for the transient MOC-IHC synapse (Zorrilla de San Martín et al., 2010), BK channels are also functionally expressed at the MOC synaptic terminals innervating the OHCs. So far, results from *in vivo* experiments with slo^−/−^ mice, which lack the BK alpha subunit, suggest that in adult mice, BK channels are not expressed by the efferent presynaptic terminals contacting the OHCs (Maison et al., 2013a). The authors argue that if BK channels were functionally expressed by the efferent terminals contacting OHCs, the knockout phenotype should show increased efferent effects, due to increased ACh release at the MOC-OHC synapse as shown at the MOC-IHC synapse (Zorrilla de San Martín et al., 2010). However, Slo^−/−^ mice were found to be characterized by reduced efferent effects (Maison et al., 2013a) which is more consistent with the postsynaptic role of BK channels at the MOC-OHC synapse (Wersinger et al., 2010). It can be misleading, however, to directly compare results from *in vitro* synaptic physiology with results obtained from *in vivo* experiments as those used to evaluate the MOC efferent effects. Notwithstanding, one can also argue that the lack of BK channels during development might have altered synaptic strength at the MOC-OHC synapse by changing the balance between regulatory mechanisms, namely GABA acting through presynaptic GABA_B_ receptors (Wedemeyer et al., 2013), the coupling of L-type VGCC to BK channels (Zorrilla de San Martín et al., 2010) as well as NO released from the postsynaptic cell upon efferent stimulation (Kong et al., 2013). Moreover, the size of the efferent terminals in the slo^−/−^ mice is reduced throughout the cochlear spiral (Maison et al., 2013a), this suggests that the lack of BK channel functional expression alters the normal MOC-hair cell synapse development and could also explain in part the reduced magnitude of efferent suppression in these mice. BK channels have been shown, at the light-microscopic level, to be expressed at the interface between presynaptic MOC terminals and the postsynaptic OHC membrane (Hafidi et al., 2005; Engel et al., 2006; Wersinger et al., 2010; Maison et al., 2013a), suggesting a postsynaptic localization for these calcium-activated K^+^ channels. However, an electron microscopic study carried out in adult mice shows by immunogold labeling that at the MOC-OHC synapse, BK channels are localized at both pre and postsynaptic membranes (Sakai et al., 2011). Therefore, in order to determine whether BK channels are functionally expressed by the MOC synaptic terminals contacting OHCs, it is necessary to evaluate the strength of synaptic transmission by electrophysiological experiments in wild-type mice in the presence of specific blockers of these channels both during development and adulthood.

#### Inhibition of ACh release by the GABAergic system

Medial olivocochlear synapses are mainly cholinergic, however, a profuse GABAergic innervation has been described close to the IHC and OHC regions (Fex and Altschuler, 1986; Vetter et al., 1991; Eybalin, 1993; Maison et al., 2003). In addition, it has been shown that GABA co-localizes with ACh in almost all efferent terminals of the OC system in adult mice (Maison et al., 2003). Experiments carried out *in vivo* with mice lacking different GABA_A_ receptor subunits presented cochlear dysfunction and suggest that the GABAergic component of the OC system contributes to the long-term maintenance of hair cells and superior cervical ganglion (SCG) neurons in the inner ear (Maison et al., 2006). Furthermore, the phenotypic analysis of GABA_B1_ knockout mice indicated that GABAergic signaling might be required for normal OHC amplifier function (Maison et al., 2009). Moreover, OHC stiffness and motility have been shown to be sensitive to exogenously applied GABA (Gitter and Zenner, 1992; Batta et al., 2004). Guinea pig OHCs have been shown to hyperpolarize upon GABA application to the extracellular medium (Gitter and Zenner, 1992) which suggests that those cells might express GABA_A_ postsynaptic receptors. However, postsynaptic GABA-activated currents were not found either in mouse IHCs (P9–11) or OHCs (P12–16) indicating that postsynaptic GABA_A_ receptors if present, are not functional in the organ of Corti of developing mice (Wedemeyer et al., 2013). Two other evidences support the notion that fast synaptic transmission at the MOC-hair synapse is cholinergic and mediated only by the postsynaptic α9α10 nAChR: (1) no postsynaptic currents are observed in the OHCs and the IHCs in response to either K^+^ elevation or electrical stimulation of the MOC efferent axons if the α9α10 nAChR is pharmacologically blocked (Glowatzki and Fuchs, 2000; Oliver et al., 2000; Ballestero et al., 2011); and (2) no postsynaptic currents are observed in α9 knockout mice (Vetter et al., 2007).

Interestingly, using pharmacological and electrophysiological approaches, together with mutant mouse lines lacking specific GABA_B_R subtypes, a physiological role for GABA in MOC efferent synaptic transmission has been recently demonstrated (Wedemeyer et al., 2013). Activation of presynaptic GABA_B_Rs by baclofen, a selective GABA_B_Rs agonist, inhibits the release of ACh from OC-IHC terminals in P9–11 mouse cochlear explants. Moreover, incubation with a selective GABA_B_R antagonist, CGP35348, significantly increases the quantum content of evoked release, demonstrating the presence of pre-synaptic GABA_B_ receptors. Furthermore, inhibition of transmitter release by GABA at the MOC-IHC synapse is most likely mediated through inhibition of P/Q- but not N-type VGCCs (Wedemeyer et al., 2013). In addition, CGP35348 also enhances evoked release at the MOC-OHC synapse in P9–16 mice, suggesting that presence of functional GABA_B_ receptors at this synapse as well. The dimerization of two subunits, GABA_B1_ and GABA_B2_, is required to make up functional GABA_B_Rs (Jones et al., 1998; Kaupmann et al., 1998; White et al., 1998; Kuner et al., 1999). The molecular variability of GABA_B_R is due to the existence of two different GB1 isoforms, 1a and 1b (Bettler et al., 2004). By making use of GABA_B_ subunit specific KO mice, it was also shown that GABA acting on presynaptic GABA_B(1a,2)_R inhibited the release of ACh at the MOC-IHC synapse (Wedemeyer et al., 2013). Immunostaining experiments in transgenic GABA_B1_-GFP mice, further demonstrated the expression of GABA_B_Rs in OC terminals innervating both IHCs and OHCs during development (P9–16). Those results are consistent with evidence indicating that the GB1a isoform is mainly expressed by the presynaptic terminals whereas the GB1b is usually found at the postsynapse (Perez-Garci et al., 2006; Vigot et al., 2006). Therefore, GABA released at both the MOC-IHC and MOC-OHC cholinergic inhibitory synapses activates presynaptic GABA_B_Rs that inhibit the release of ACh thus reducing inhibition. This agrees with the widely described role of GABA in presynaptic modulation of synaptic transmission at mammalian glutamatergic and GABAergic synapses (Gaiarsa et al., 1995a,b; Brenowitz et al., 1998; Chalifoux and Carter, 2011a,b).

Immunohistochemistry experiments have revealed that both GABA_B_ receptors and GAD, the GABA synthetic enzyme, are present in the synaptic terminals contacting both IHCs and OHCs close to hearing onset (Wedemeyer et al., 2013). This is consistent with previous data showing that the GABAergic input to the mammalian cochlea arises solely from the OC system (Fex and Altschuler, 1986; Thompson et al., 1986; Vetter et al., 1991; Eybalin, 1993; Maison et al., 2003). However, in adult mice, GABA_B_Rs have not been found in OC efferent terminals making synaptic contacts in the IHC or the OHC regions (Maison et al., 2009), a result that might indicate that presynaptic modulation of ACh release by GABA_B_ receptors is limited to the developing MOC system. Adult IHCs are not innervated by the MOC system (Liberman, 1980; Simmons, 2002), so the lack of GABA_B_Rs at the IHC area is not surprising. However, OHCs are innervated by MOC fibers since the second postnatal week throughout life (Liberman, 1980; Simmons, 2002). Expression of functional GABA_B_Rs in MOC fibers innervating the OHCs (Wedemeyer et al., 2013) might be transient, and this needs to be further explored. Alternatively, GABA_B_ receptors might be present in adult OHC efferent terminals but the expression level might be below that detected by immunostaining methods.

GABA and ACh were reported to be co-localized in the same MOC terminals (Maison et al., 2003). Therefore, it is likely that both neurotransmitters are co-released from the same terminal when the efferent fibers are activated. Co-release of these two neurotransmitters was described in retinal starburst amacrine cells, but in this case both GABA and ACh act at postsynaptic receptors (Duarte et al., 1999; Lee et al., 2010). At the OC-hair cell synapse, however, ACh release is modulated by putatively co-released GABA by activating GABA_B_ autoreceptors. Presynaptic inhibition of ACh release via GABA_B_Rs might be involved in shaping the short term plasticity properties of the MOC-hair cell synapses (see Section Short-term Plasticity at Olivocochlear Synapses).

#### Retrograde enhancement of ACh release by nitric oxide

It has been shown recently that nitric oxide (NO), probably released by the IHCs in response to low frequency stimulation (1 Hz) of the efferent MOC fibers, increases the efficacy of transmitter release at the MOC synaptic terminals acting as a retrograde signal (Kong et al., 2013). Both IHCs and OHCs can produce NO in response to ATP-evoked calcium influx (Shen et al., 2006), and NO synthase immunoreactivity has been described throughout the cochlear epithelium, including hair cells, and afferent and efferent nerve endings (Heinrich et al., 1997; Riemann and Reuss, 1999). It still remains to be elucidated however, the mechanism by which NO enhances release. Nitric oxide stimulates guanylate cyclase to produce cyclic GMP, leading to cGMP-dependent phosphorylation of vesicular release proteins and thus might alter the synaptic protein interactions that regulate neurotransmitter release and synaptic plasticity (Meffert et al., 1996). In addition, NO might alter channel gating by direct nitrosylation of the channel protein (Bredt and Snyder, 1994). Therefore, it would be interesting to evaluate whether NO enhances the activity of either P/Q and/or N-type VGCCs or if it interferes with the negative feedback loop involving functionally coupled L-type VGCCs and BK channels described at the MOC-IHC transient synapse (Zorrilla de San Martín et al., 2010).

## Short-term synaptic plasticity

Synapses are endowed with an extraordinary capacity to change according to their previous history. Several forms of activity-dependent synaptic plasticity shape synaptic output (Zucker and Regehr, 2002). Short-term plasticity (STP) lasts from tens of milliseconds to several minutes and can modify synaptic strength, which can be reduced for hundreds of milliseconds to seconds (depression), or it can be enhanced for hundreds of milliseconds to seconds (facilitation), to tens of seconds to minutes (augmentation and post-tetanic potentiation, PTP). The interaction between multiple forms of plasticity will lead to the observed net plasticity at any given synapse. In many cases, facilitation, depression, PTP and longer lasting depression co-exist, but the relative salience of each mechanism is controlled by the initial release probability and the presynaptic activity pattern (Zucker and Regehr, 2002; Fioravante and Regehr, 2011). Synapses with a high probability of release tend to depress, whereas those with a low probability of release usually facilitate when challenged by two closely spaced stimuli (Fioravante and Regehr, 2011). The origin of STP is thought to be mainly of presynaptic origin, although under certain conditions postsynaptic mechanisms have been shown to be involved (Dittman et al., 2000; Schneggenburger et al., 2002; Wang and Manis, 2008). Regulation of VGCCs has not been generally considered as a major mechanism in STP, however it has been recently shown that regulation of VGCC can mediate STP (Catterall and Few, 2008; Catterall et al., 2013). There is accumulated evidence indicating that facilitation is caused by an elevated intracellular Ca^2+^ concentration that remains from the previous stimulus. Residual Ca^2+^ is thought to increase the release probability by binding to a Ca^2+^ sensor different from the one that mediates evoked exocytosis (Schneggenburger et al., 2002). Short-term synaptic depression upon high frequency stimulation is thought to arise due to depletion of a readily releasable pool of vesicles (RRP; Atluri and Regehr, 1996; Neher, 1998; Schneggenburger et al., 2002).

### Short-term plasticity at olivocochlear synapses

The main function of the vertebrate efferent system is well conserved among species. Namely, it inhibits the activity of hair cells and thereby regulates the dynamic range of hearing (see Guinan, 2011). A low probability of release at rest and facilitation of responses during high-frequency discharges has been reported in the turtle papilla (Art et al., 1984), at the transient MOC efferent synapse to mammalian IHCs before the hearing onset (Goutman et al., 2005) and more recently also at the MOC-OHC synapse (Ballestero et al., 2011). These well conserved synaptic mechanisms suggest they are relevant for regulating auditory function.

In rat P9–11 MOC-IHC synapses, transmitter release increases upon high frequency stimulation (40 Hz) of the MOC fibers due to presynaptic facilitation and postsynaptic summation (Goutman et al., 2005). Surprisingly, the same stimulation pattern, and using the same cochlear preparation produced depression in MOC-IHC synapses from P9–11 mice (Zorrilla de San Martin et al., 2012). The quantum content of ACh release at both synapses was found to be similar and low (around 1), so other factors (i.e., size or rate of replenishment of the readily releasable pool of vesicles (RRP), the Ca^2+^ channels supporting and/or modulating release) might account for this species differences.

**Figure 4:**
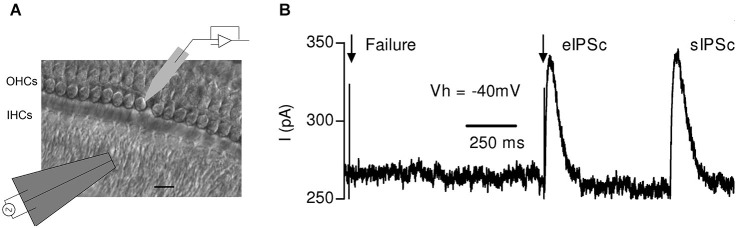
**Synaptic responses evoked by electrical stimulation of the MOC efferent fibers in mouse OHCs. (A)** Schematic representation of the experimental setup: OHCs from the first row were recorded with a patch pipette while electrical shocks were delivered to the MOC fibers through an extracellular bipolar theta glass pipette positioned ~10–20 μm below the IHCs. Scale bar 10 μm.**(B)** Representative trace obtained in an OHC in response to two single electrical shocks (arrows). The figure shows one evoked and one spontaneous inhibitory postsynaptic current (eIPSC) and (sIPSC), respectively, and also a failure of response upon nerve stimulation. (Reproduced with permission from Figure 1 in Ballestero et al., 2011).

**Figure 5:**
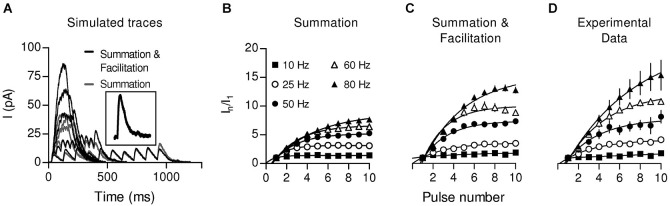
**Summation and Facilitation contribute to the increment of the postsynaptic response during high frequency stimulation**. To estimate the effect of summation during high frequency stimulation, simulated responses were constructed from a single-shock response (**A**, inset) considering only temporal summation (gray traces) or considering facilitation by also taking into account the change in the probability of release (black traces) for every shock. Plots of normalized current vs. pulse number were constructed from the simulation considering only summation **(B)** or summation and facilitation **(C)**. **(D)** Plot of normalized current vs. pulse number derived from the experimental data. (Reproduced with permission from Figure 7 in Ballestero et al., 2011).

By performing whole-cell recordings in voltage-clamped mouse OHCs while electrically stimulating the MOC efferent fibers innervating them (Figure 4), Ballestero et al. (2011) showed how synaptic transmission is tuned at the MOC-OHC synapse. At a stimulation frequency of Hz transmitter release at the MOC-OHC occurs with low probability (quantum content ~0.4). When the stimulation frequency is raised, the efficacy of release increases due to presynaptic facilitation. In addition, the relatively slow decay of evoked inhibitory postsynaptic currents (eIPSCs) (combined nAChR and SK2 IPSCs; see Figure 1) causes temporal summation at frequencies >10 Hz. Facilitation and summation give rise to a frequency-dependent increase in the amplitude of inhibitory currents in OHCs (Figure 5; Ballestero et al., 2011).

The properties of the MOC-OHC synapse described by Ballestero et al. (2011) are consistent with *in vivo* studies performed to evaluate the MOC efferent effects on hearing. Electrical stimulation of MOC neurons only inhibits auditory function efficiently when high frequency trains are applied (Galambos, 1956; Wiederhold and Kiang, 1970; Mountain, 1980; Gifford and Guinan, 1987). Moreover, the strength of the efferent effect increases linearly upon increasing the stimulation rate, not only in mammals (Galambos, 1956; Wiederhold and Kiang, 1970; Brown and Nuttal, 1984; Gifford and Guinan, 1987) but also in other vertebrates (Flock and Russell, 1976; Art et al., 1984; Figure 12A). In addition, MOC firing rate increases with sound intensity (Robertson and Gummer, 1985; Brown, 1989; Brown et al., 1998) leading to a greater gain reduction upon exposure to intense sounds. This is in agreement with the hypothesis that the MOC system protects the auditory system from acoustic trauma (Rajan, 2000; Taranda et al., 2009).

Efferent synaptic terminals from various species were shown by electron microscopy studies to have a large number of synaptic vesicles (Lenoir et al., 1980; Nadol, 1988; Simmons et al., 1996; Bruce et al., 2000). Therefore, the low probability of release at the MOC-OHC synapse at low frequency stimulation (Ballestero et al., 2011) cannot be accounted for by vesicle availability (Schikorski and Stevens, 1997; Xu-Friedman and Regehr, 2004). Moreover, the fact that synaptic output at the MOC-OHC synapse can be sustained during prolonged periods even at high stimulation frequencies (Ballestero et al., 2011), gives further support to the hypothesis that vesicle availability is not the limiting factor.

**Figure 6:**
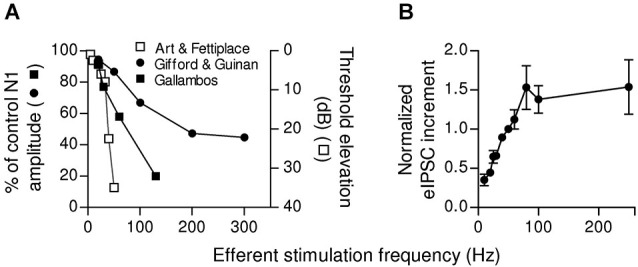
**Dependence of afferent function and OHCs inhibitory responses on the frequency of efferent stimulation. (A)** Relationship between the frequency of MOC activation and inhibition of afferent activity in the cat (adapted from Galambos, 1956; Gifford and Guinan, 1987) and in the turtle (adapted from Art and Fettiplace, 1984). In the left y-axis efferent effect was quantified as the ratio between the amplitude of the N1 component of the compound action potential (CAP) produced by moderate sound stimulation (10–25 dB above threshold) with or without efferent stimulation ((N1_w/shock_/N1_ctrl_)x100, (Galambos, 1956; Gifford and Guinan, 1987). The right y-axis shows the increase in sound intensity (Threshold shift in dB) necessary to evoke an afferent discharge as a function of the efferent stimulation frequency (Art and Fettiplace, 1984). **(B)** Increment in eIPSC amplitudes (mean ± SEM) after 10 shocks of efferent stimulation at different frequencies. Due to data variability, the values from different cells were normalized to the increment in the amplitude of the response at a stimulation frequency of 50 Hz. Note that in both afferent activity **(A)** and OHCs eIPSCs **(B)**, there is a range of frequencies where there is a linear relationship between the frequency of MOC activation and the increment in inhibition. (Reproduced with permission from Figure 12 in Ballestero et al., 2011).

Some synapses have a low initial release probability and present strong facilitation. They respond with high efficacy only to high frequency stimulation (Lisman, 1997) and function as “high-pass filters” (Fortune and Rose, 2001). This mechanism, which implies that spontaneous or infrequent MOC discharges are ignored, would determine a threshold for efficient cochlear suppression. In mammals, this could be of great relevance as it was shown that MOC fibers fire regularly but with variable frequencies (Robertson and Gummer, 1985; Liberman and Brown, 1986; Brown, 1989). Moreover, summation and facilitation at the MOC-OHC synapse can grade efferent inhibition according to MOC discharge rate, thus fine tuning cochlear amplification. The firing rate of MOC fibers increases linearly with sound intensity (Robertson and Gummer, 1985; Liberman and Brown, 1986; Brown, 1989). Besides, upon activation by sound MOC fiber discharge rate is modulated by stimulus properties such as intensity, origin, and type (Brown et al., 1998). In this scenario, the short term plasticity properties of the MOC-OHC synapse seem to be highly relevant for encoding graded levels of efferent feedback. This has been illustrated in Figure 6, where it is shown that the reduction in auditory brain response amplitude upon increasing the frequency of MOC stimulation (Galambos, 1956; Art and Fettiplace, 1984; Gifford and Guinan, 1987) is in agreement with the increment in the amplitude of OHC synaptic responses when the frequency of stimulation of the MOC efferent fibers is increased (Ballestero et al., 2011).

## Developmental molecular and functional changes at the MOC-IHC synapse

As described above, IHCs are transiently innervated by fibers of the MOC system which make functional axo-somatic contacts with these cells since birth until the onset of hearing (P12 in mice and rats) (Glowatzki and Fuchs, 2000; Simmons, 2002; Katz et al., 2004; Roux et al., 2011). During development, synaptic modifications take place concurrently in both postsynaptic cells and presynaptic terminals. The transient MOC-IHC synapse is no exception and it undergoes dramatic changes both in the cholinergic sensitivity, the expression of key postsynaptic molecules (Katz et al., 2004; Marcotti et al., 2004; Roux et al., 2011), as well as in the pattern of innervation (Simmons, 2002; Katz et al., 2004; Roux et al., 2011). Thus, the expression of the nAChR α10 subunit and the SK2 channel are down regulated and disappear after the onset of hearing (Katz et al., 2004). This is accompanied by a retraction of the axo-somatic contacts to these cells (Simmons, 2002). Even though, the mRNA for the α9 subunit is present in IHCs throughout life (Elgoyhen et al., 1994, 2001), no cholinergic responses can be found in these cells after the onset of hearing (Katz et al., 2004; Roux et al., 2011). Cholinergic responses at P0 are excitatory as they are not coupled to the SK2 channel (Roux et al., 2011) and they dramatically increase from P1 towards P7–9, they start to decline at around P12 to completely disappear after P14, consistent with the down regulation of the expression of the α10 subunit and the SK2 channel (Katz et al., 2004; Roux et al., 2011).

Ca^2+^ channels coupled to transmitter release are developmentally regulated both at central synapses (Momiyama, 2003; Fedchyshyn and Wang, 2005) and at the neuromuscular junction (Rosato Siri and Uchitel, 1999). In agreement with this notion, it has been recently shown that there are significant changes in the types VGCC that support and/or modulate the release process at the MOC-IHC synapse during the short period at which it is functional. Namely, at P9–11, P/Q and N-type VGCC support release whereas Ca^2+^ through L-type VGCC activate BK channels (Zorrilla de San Martín et al., 2010). At P5–7, N-type channels are not functionally expressed by the transient MOC synaptic terminals and transmitter release is supported by P/Q and R-type VGCC (Zorrilla de San Martin et al., 2012; Kearney et al., 2014). Moreover, at P5–7, L-type VGCC, both support release and activate BK channels (Kearney et al., 2014), indicating that at an earlier stage of development, the presynaptic terminal might be less compartmentalized than at P9–11 (Zorrilla de San Martin et al., 2012). Moreover, it has been recently shown that at this earlier stage of development (P5–7), the mouse MOC-IHC synapse presents a lower initial probability of release than that at P9–11 and that it facilitates upon high frequency stimulation (10-pulse trains at 40 and 100 Hz lead to ~2-fold increase in synaptic efficacy), whereas the same stimulation protocol applied at P9–11 synapses leads to progressive depression (Zorrilla de San Martin et al., 2012). Thus, the changes in expression of key molecules involved in synaptic transmission that take place both in the pre and the postsynapse at the transient MOC-IHC synapse (Katz et al., 2004; Roux et al., 2011; Zorrilla de San Martin et al., 2012; Kearney et al., 2014) during the short period at which it is functional, might underlie the changes in the STP properties of this synapse (Zorrilla de San Martin et al., 2012). In addition, developmental changes in STP properties of the transient MOC-IHC synapse might lead to fine tuning of the pattern of action potential frequency of IHCs (Goutman et al., 2005; Johnson et al., 2011; Sendin et al., 2014), affecting signaling at the first synapse of the auditory system during the course of its establishment.

Interestingly, it has been very recently demonstrated that the normal strengthening and silencing of inhibitory synaptic connections between the medial nucleus of the trapezoid body (MNTB) and the LSO before hearing onset is impaired in mice lacking a functional efferent innervation, the α9 KO mice (Clause et al., 2014). It is important to mention that MNTB neurons discharge following the spike patterns of SGN (Tritsch and Bergles, 2010; Tritsch et al., 2010) which are activated by glutamate release at the IHC-afferent fiber synapse. This release of glutamate occurs in the absence of sensory stimuli, before hearing onset, and is driven by Ca^2+^ action potentials in IHCs (Beutner and Moser, 2001; Glowatzki and Fuchs, 2002). Moreover, α9 nAChR subunit KO mice have severe deficits in the axonal pruning that occurs in normal mice during the first week after the onset of hearing (Clause et al., 2014). In addition, it has been also recently shown that the Ca^2+^ sensitivity of glutamate release at afferent IHC-ribbon synapses do not mature correctly in the α9 nAChR subunit KO mice (Johnson et al., 2013). The above mentioned results show that the timing and pattern of activity that occurs in the absence of sensory stimuli before hearing onset, is important for the correct development of a tonotopic map. In addition, these results give strong support to the hypothesis that efferent MOC modulation of the IHC action potential pattern (Glowatzki and Fuchs, 2000; Marcotti et al., 2004; Goutman et al., 2005; Johnson et al., 2011; Sendin et al., 2014) is a key factor in the correct establishment of the auditory pathway. Thus it can be hypothesized that the transient efferent innervation to IHCs could be involved in the functional maturation of IHCs, as well as in the correct development of the peripheral and central compartments of the auditory system.

## Conclusions

Synaptic strength is a key variable for transmitting information, therefore synapses, both in the developing and mature nervous system, must be highly regulated in order to adapt to the changing demands of the environment. The MOC-hair cell synapse is endowed with at least three regulatory mechanisms: presynaptic inhibition of ACh release via the activation of BK channels that reduce action potential duration (Zorrilla de San Martín et al., 2010); inhibition of ACh release via GABA_B_Rs reducing Ca^2+^ entry through P/Q-type VGCC (Wedemeyer et al., 2013) and enhancement of ACh release by a retrograde messenger, most likely, NO (Kong et al., 2013). These regulatory mechanisms acting in concert (see Figure 3) might determine the STP properties of MOC-hair cell synapses and thereby exert a tight control on the release probability. As reported for other synapses (Brenowitz et al., 1998; Brenowitz and Trussell, 2001), this regulation could be crucial for preventing depression and allow MOC fibers to continue releasing transmitter even at high activity rates. Moreover, as MOC firing rate increases with sound intensity (Brown et al., 1985; Robertson and Gummer, 1985) and this increment in firing rate enhances the efficacy of the MOC-OHC synapse (Ballestero et al., 2011), synaptic plasticity might be relevant for protecting the auditory system from noise-induced damage (Rajan, 2000; Maison et al., 2002, 2013a; Wang et al., 2002). Finally, since the efficacy of the MOC efferent innervation to diminish or silence spontaneous Ca^2+^ action potentials in IHCs improves as the MOC firing frequency increases (Goutman et al., 2005), STP might also be crucial for regulating the activity pattern of the first afferent synapse during the establishment of the auditory pathway.

## Conflict of interest statement

The authors declare that the research was conducted in the absence of any commercial or financial relationships that could be construed as a potential conflict of interest.
